# Potential and Challenges of Community-Based Surveillance in Animal Health: A Pilot Study Among Equine Owners in Switzerland

**DOI:** 10.3389/fvets.2021.641448

**Published:** 2021-06-04

**Authors:** Ranya Özçelik, Franziska Remy-Wohlfender, Susanne Küker, Vivianne Visschers, Daniela Hadorn, Salome Dürr

**Affiliations:** ^1^Veterinary Public Health Institute, Vetsuisse Faculty, University of Bern, Bern, Switzerland; ^2^ISME Equine Clinic Bern, Vetsuisse Faculty, University of Bern, Bern, Switzerland; ^3^School of Applied Psychology, University of Applied Sciences and Arts Northwestern Switzerland, Olten, Switzerland; ^4^Federal Food Safety and Veterinary Office, Bern, Switzerland

**Keywords:** equine, animal health surveillance, equine owner, transdisciplinary, surveillance, community, surveillance system, community-based surveillance

## Abstract

Animal owners' potential to observe and report clinical signs, as the persons with the closest contact to their animals, is an often neglected source of information in surveillance. Allowing community members other than health care professionals, such as animal owners, to report health events can contribute to close current surveillance gaps and enhance early detection. In the present study, we tested a community-based surveillance (CBS) approach in the equine community in Switzerland. We aimed at revealing the attitudes and intentions of equine owners toward reporting clinical signs by making use of an online questionnaire. We further set up and operated an online CBS tool, named Equi-Commun. Finally, we investigated potential reasons for the lack of its use by applying qualitative telephone interviews. The majority of the respondents of the online questionnaire (65.5%, 707/1,078) answered that they could see themselves reporting clinical observations of their equine. The multivariate logistic regression analysis indicated that French-speaking equine owners and those belonging to the positive attitude cluster are more likely to report to a CBS tool. Equi-Commun operated between October 2018 and December 2019 yet received only four reports. With the addition of qualitative interviews, we identified three critical, interlinked issues that may have led to the non-use of Equi-Commun within the Swiss equine community: (1) for successfully implementing CBS, the need for surveillance within the community of interest must be given; (2) the respective population under surveillance, here the equine, needs to show enough clinical cases for owners to be able to maintain the memory of an existing tool and its possible use; and (3) targeted and high effort communication of the system is key for its success. While CBS relying only on lay animal owners, complementary to existing surveillance systems, could potentially provide a good proxy of timely surveillance data, it is questionable whether the added value of generated surveillance knowledge is in balance with efforts necessary to implement a successful system. With this study, we showcased both the potential and challenges of CBS in animal health, as this may be of relevance and guidance for future initiatives.

## Introduction

Animal health surveillance has been developing continuously over the past decades, providing new concepts, approaches, and methods for improvement and refinement of animal health (1–3). Health professionals, such as veterinarians, play a crucial role in surveillance. They are involved in routinely collecting animal health and production data, such as for syndromic surveillance and active surveillance programs, as well as in providing necropsy reports and diagnostic laboratory data (3–8). Surveillance data, except for syndromic surveillance, predominantly depend on clinical cases being presented to health professionals (9). Yet not all diseased humans or animals seek–or are brought to receive–medical care, for reasons such as concern about health care costs and the individual person's perception of a certain clinical case being severe enough to be presented to a health care professional (10–13). Meanwhile, animal owners' potential to observe clinical signs, as the persons with the closest contact to their animals, is often neglected. Inclusion of animal owners in animal health surveillance, in the frame of community-based surveillance (CBS), could complement and strengthen existing surveillance efforts.

Up to date, the term CBS has mainly been used in the context of public health and One Health surveillance, while predominantly implemented in low- and lower-income countries (14). The World Health Organization (WHO) defined the term CBS in the context of public health as follows: “CBS is the systematic detection and reporting of events of public health significance within a community by community members.” (15). CBS has been shown to have the potential to close surveillance gaps by complementing existing surveillance systems, especially in settings where logistic or socio-cultural factors for accessing certain populations and generating data are limited or personnel and financial resources are tight (14, 16, 17). As a recent example, a CBS tool provided by the International Red Cross and Norwegian Red Cross (https://www.cbsrc.org/) organizations allowed the detection of the first coronavirus disease 2019 (COVID-19) case in Somaliland (18).

While an official definition and uniform use for the term CBS in animal health is lacking, there are various approaches and initiatives making use of community members' involvement and engagement in animal health surveillance. Such approaches are embedded in participatory surveillance, participatory epidemiology, citizen science, and owner-based reporting of health-related data (19–22). As an example, a CBS system established up-on community animal health workers in Tanzania has previously shown to enhance surveillance capacity by increasing spatial coverage of surveillance systems and deliver timely data on community-based disease observations in livestock (23). Whether it is by deploying trained staff or by lay people, CBS can contribute to close current surveillance gaps and enhance early detection by allowing community members other than designated health care professionals to report and alert for health events (24). Although CBS systems in animal health are predominantly described from low- and middle-income countries, examples of systems collecting animal health data by owners also exist in developed countries. In many European countries, farmers are obliged to systematically record diverse health data of their livestock, such as antimicrobial use, abortions, and deaths (25–27). However, many such systems are only in place because of binding regulations and laws, therefore are not based on voluntary compliance of animal owners.

Within the field of equine health, multiple studies have described the use of owner-based reports to limit knowledge gaps of certain health disorders. In a cross-sectional survey in Australia, equine owners' capacity to observe the health of their own equine was used to determine the prevalence of a wide range of health disorders (28). Likewise, in Great Britain a survey was performed among equine owners over a 2-year period on preventive health care measures and certain disease prevalence of their equine (29). In another study from Great Britain, equine owners were asked to report laminitis episodes through a web-based form for an overall study period of 2 years to overcome the suspected underestimation of veterinary-diagnosed equine laminitis incidences (21). While each of these studies made use of equine owners' capability to observe and report clinical signs, they have in common that reporting was temporally limited. They thus only allowed a cross-sectional insight to current disease events based on the knowledge and observations of equine owners, however did not provide continuous surveillance. The French epidemiological network for equine diseases (Réseau d'Epidémio-Surveillance en Pathologie Equine, “RESPE”) announced the implementation of the project VigiRespe, which was created to complement existing veterinary surveillance data and increase sensitivity with the help of observations by equine owners (30–32). Yet peer-reviewed publications regarding their experiences on the system are lacking up to date. Thus, to the best of our knowledge, attempts to include non-sentinel and voluntary equine owners for continuous disease surveillance have not been investigated yet.

The surveillance of equine health in Switzerland currently includes the mandatory reporting of 17 notifiable diseases according to the Swiss animal health law (Federal Council of Switzerland, 1995). In addition, Equinella (www.equinella.ch), a veterinary-based voluntary surveillance system for clinical signs and equine diseases not notifiable by the Swiss law, is in place (33–35). During a presentation on Equinella at the 2016 Swiss annual scientific conference and network of equine health research, equine practitioners as well as various stakeholders from the Swiss equine industry arouse the question whether equine owners could also contribute to Equinella. This input from the equine community showed the interest to participate in surveillance systems and was one of the drivers of the here presented CBS approach.

In the present study, we aimed to reveal the perception, attitude, and intention of Swiss equine owners toward CBS and report clinical signs of their equine. First, a cross-sectional online questionnaire was sent to Swiss equine owners. Second, we set up and operated an online CBS tool, named Equi-Commun. The aim of Equi-Commun was to assess the benefits of surveillance data derived from equine owners compared with already existing surveillance data from Equinella in terms of timeliness of reporting, as well as data quantity and quality. It, however, received only four reports during the 1-year pilot phase and was put to rest due to non-use. We, therefore, finally investigated potential reasons for the lack of its use by applying qualitative telephone interviews among equine owners. In summary, we present and discuss the potential and challenges of CBS as well as possible reasons for the lack of compliance of equine owners to a CBS system, which may be informative for animal health surveillance systems beyond equine health.

## Materials and Methods

### Online Survey

The online survey aimed at revealing the perception, attitudes and intention of equine owners in Switzerland toward CBS and toward reporting clinical signs of their equine, as well as determining factors (positive and negative beliefs and demographic parameters) influencing these attitudes and intentions. In addition, information on clinical signs in equines discovered by their owners during the last 12 months was interrogated to generate a baseline value on cases that may be reported to the CBS system.

#### Questionnaire Design and Launch, and Data Export

The questionnaire (Supplementary Material 1) was developed by a transdisciplinary team of epidemiologists, veterinarians, Veterinary Public Health specialists, and a psychologist. It contained 31 questions, of which 19 were mandatory, including single response, multiple choice, 5- and 6-point Likert scale, and free-text questions, embedded in four main parts; (1) information on equine owned and/or kept on the own premises, (2) clinical signs observed among their equine and/or the equine kept on the own premises within the last year, (3) intention to use and attitude toward a proposed CBS tool and, (4) demographic data of the respondent. To enable participants to reflect on the questions regarding their attitude toward a CBS tool, a description of the not yet launched “Equi-Commun” was provided within the questionnaire (Supplementary Material 1, section C, question 16). The questionnaire was designed in German and later translated into French and Italian. Once finalized, the survey was programmed in LimeSurvey (https://www.limesurvey.org/) and pre-tested by 24 equine owners in all three languages and thereafter adapted according to their feedback. Persons registered to the Agate portal (36) (mandatory register for equine owners in Switzerland), owning or accommodating at least one equine (horses, ponies, donkeys, or mules), and having an e-mail address build the sampling frame (~53,000 equine owners). A sample of 7,500 equine owners was randomly retrieved from the sampling frame. This sample size was calculated using a design prevalence of 50% (to provide the largest samples size), a confidence level of 95%, a precision rate of 5%, and an estimated response rate of 20%. The questionnaire was sent out as a link within an e-mail to all 7,500 recipients on July 11, 2018 by the Food Safety and Veterinary Office (FSVO). Participants were offered to leave their e-mail address at the end of the questionnaire to be informed about future steps regarding the project. A reminder e-mail for the online questionnaire was sent to all recipients after 1 week. The survey was accessible for 2 weeks in total. Data were exported from LimeSurvey in Microsoft Excel for data analysis. According to Swiss legislation, studies that do not collect sensitive human personal data nor human health-related information do not require an ethical approval. This also applies to the telephone interviews (Qualitative Interviews section).

#### Data Analysis

Only fully completed questionnaires were taken into account for the statistical analysis. The analyses were conducted in R statistical software version 3.6.2 (37). Descriptive statistics on the study population and the intention of equine owners to report to the CBS system was performed.

The attitude toward CBS was assessed using three questions from the survey, two Likert scales and one multiple choice questions (questions 21, 22, and 23 in the questionnaire, Supplementary Table 1). Factors captured by these questions covered positive and negative beliefs, such as the perceived value of equine health, incentives for participation in the form of free information or economic benefits, and reasons for not wanting to participate. We conducted a Multiple Component Analysis (MCA) to group respondents according to the answers to these three questions using the R package FactoMineR (38). Results from the MCA were used to classify respondents into hierarchical clusters (further referred to as “attitude cluster”) using the Hierarchical Clustering on Principal Components (HCPC) function of the FactoMineR package. The variable *attitude cluster* was used as an explanatory variable in the logistic regression analysis.

One question from the survey was used to evaluate the self-reported intention to contribute to the CBS tool (question 18 in the questionnaire, Supplementary Material 1). This question was asking whether respondents could imagine themselves reporting to a CBS tool. The responses to the 5-point Likert scale of this question were transformed into a binary factor by aggregating “certainly yes,” “presumably yes,” and “maybe” as positive answers (707/1,078 answers) versus “certainly no” and “presumably no” (371/1,078 answers) as negative answers. This binary outcome was used as the outcome variable (*would report*) in a logistic regression analysis investigating factors influencing the intention to contribute to a CBS tool.

First, univariate logistic regression models were built with *age, gender, type of ownership, type of premise, sum of clinical signs observed during the past year, profession, language, frequency of visiting the equine, transport of equine*, and *attitude cluster* being explanatory variables, whereas *would report* was selected as the outcome variable (Table 1). Second, multivariable regression models were built with variables associated with a *p* < 0.2 in the univariate regression models. The final model was identified by stepwise backwards selection of the explanatory variables choosing the model with the lowest Akaike's Information Criteria (AIC) as selection criteria. Variables with coefficient *p*-values of <0.05 were considered as statistically significant in the final multivariable model.

**Table 1:** Description of the characteristics of equine owners from fully completed questionnaires, *n* = 1,078.

**Variable**	**Category**	**Value (n)**	**Percentage (%)**
Age	Average	48.6	–
	Median	50	–
	IQR	39–57	–
Gender	Women	834	77.4
	Men	219	20.3
	Prefer not to say	25	2.3
Language	German	868	80.5
	French	194	18.0
	Italian	16	1.5
Type of ownership	Equine owner	604	56.0
	Equine and premise owner (both)	401	37.2
	Premise owner	73	6.8
Type of premise	Agricultural farm with equine only or equine and other livestock	558	51.7
	Equine on own private ground	295	27.4
	Equine pension premise	196	18.2
	Unknown	23	2.1
	Other (breeding establishment, animal park, training establishment)	6	0.6
Sum of clinical signs observed during	In total by all respondents	17,016	–
the past year	Median per respondent	4	–
	Range per respondent	0–340	–
	IQR	1–11	–
Attitude cluster	Highly positive attitude cluster	446	41.4
	Moderately positive attitude cluster	563	52.2
	Negative attitude cluster	69	6.4
Profession	Working with equine	160	14.8
	Human health care	165	14.5
	Animal health care	40	3.7
	Farmer	154	15.2
	I prefer not to say	138	12.8
	Other	430	39.9
Frequency of visiting the equine	Lives at the same premise	343	31.8
	Once a day at least	498	46.2
	Multiple times per week	197	18.3
	Once a week or more seldom	40	3.7
Transport of equine	Yes	598	55.5
	No	490	44.5

*These variables were used in the regression analysis to explore factors influencing the intention toward community-based surveillance (CBS) and the CBS tool Equi-Commun*.

### CBS Tool Development and Testing

The CBS tool Equi-Commun was conceptually designed following the structure of the veterinary-based voluntary surveillance system Equinella (33–35). Print-screens of the tool user interface are presented in the supplementary materials (Supplementary Figure 1). The publicly accessible online tool was technically implemented by a professional IT company (https://www.4eyes.ch/#start) and went live on October 22, 2018. Equi-Commun was addressed to equine owners [further referred to as reporting person(s)] to report observation on clinical signs of their equine as soon as they are observed. Reporting persons were given the option to choose whether or not to register to the system and, thus, create a personal login before reporting their observation. Registration came along with the advantage to access a login secured internal space with a list of previous own reports and automatic completion of information on previously registered equine (name, age, location). For each record, the following data had to be registered on Equi-Commun: name of the affected equine (manual entry), location and postal code of the equine (both manual entries yet interconnected with each other), number of equine on the premise of the reported equine (categorical list of options), observed clinical sign (at least one has to be selected from a predefined list of options), date of onset of the observed sign(s) (date selection from calendar), and duration of the observed sign(s) (categorical list of options). In addition, whether or not a veterinarian was contacted, and in case yes, who this was (manual entry), when the visit took place (date selection from calendar), and the diagnosis made by the veterinarian (manual entry) was requested as optional data. If the reporting person registered to the system for the first time, surname and e-mail address were requested as obligatory, and the primary responsible veterinarian as well as how the person knew about Equi-Commun was requested as optional data. When a record was submitted, an automatic response was generated on the website stating that the report was successfully submitted. Simultaneously, the Equi-Commun team was automatically notified on the submitted report. In general, by accepting the term and conditions stated at the end of each report submission, reporting persons were obliged to agree that the data provided can be used for research purposes in an anonymized version. An ethical approval for collecting CBS data of equines through equine owners was not necessary according to Swiss legislation.

In multiple rounds, the online tool was tested by the authors, the project supporting team of the FSVO, and the equine owners for practicability, logic, user-friendliness, and correct automatic responses. Agreed changes were later implemented by the IT Company. Equi-Commun was communicated and promoted through diverse communication strategies and multiple media channels between July 2017 and June 2019 (Supplementary Table 2). They consisted of presentations at scientific conferences; print media articles in equine magazines; distribution of flyers in equine clinics; e-mails sent to participants of the online survey to inform about the launch of Equi-Commun, to the Swiss veterinary faculty staff, and to veterinarians *via* the Equinella newsletter; and regular social media performance *via* Equi-Commun Facebook page.

### Qualitative Interviews

To assess potential reasons for the equine owners' lack of compliance toward Equi-Commun, we conducted semi-structured qualitative phone interviews. An interview guideline was drafted according to the recommendations of Helfferich (39) and based on previous knowledge collected through the online questionnaire. Interview questions focused on capturing the knowledge and understanding of equine owners in regard to CBS and the CBS tool Equi-Commun, how they came in contact with it, reasons why they did not use Equi-Commun as well as reasons they thought why other equine owners did not use Equi-Commun, and what they recommended for promoting Equi-Commun successfully (Table 2). The study population for the interviews was recruited in two steps. First, equine owners who voluntarily left their e-mail-address during online survey in July 2018 (*n* = 561) were contacted per e-mail and invited to participate in a phone interview. As a motivation for participation, a voucher from an equine tack shop (CHF 50.-) was offered. Within 11 days, 108 equine owners indicated their interest. Second, of this subpopulation, 10 equine owners were randomly selected. The phone interviews were conducted in November 2019 and recorded digitally with prior oral consent from the interviewees. The interview time lasted on average for 15 min. The recordings were digitally transcribed verbatim. The transcripts were analyzed using the qualitative data analyzing software MAXQDA2020 Analytics Pro (VERBI Software, Berlin, Germany) applying an inductive open coding approach. The inductive open coding was conducted by reading the transcripts and selecting text parts related to a certain topic mentioned by the interviewee. This approach was repeated for all transcripts, and similar textual context among different interviewee transcripts was assigned to the same code. Matching certain transcript parts to codes was repeated until all transcripts were analyzed and no new codes were identified. Conducting the interviews, transcription and coding was done by one researcher for all interviews to ensure a homogeneous view on the complete study material. For the purpose of this publication, quotes were translated from German to English and adjusted for better understanding, if grammatically necessary.

**Table 2:** Interview questions asked to equine owners during semi-structured telephone interviews on their knowledge and attitude toward Equi-Commun and on reporting clinical signs of their equine.

**Interview questions**
1) What do you know about Equi-Commun and what do you think about this platform?
2) How did you come in contact with Equi-Commun?
3) Do you feel informed about Equi-Commun?
4) Do you see any benefits in Equi-Commun? If not, why?
5) What are reasons for equine owners not reporting clinical signs of their equine?
6) Did you report any clinical signs? If not, why?
7) Do you have a suggestion what could be done differently or better for promoting Equi-Commun?

## Results

### Online Survey

#### Response Rate and Demography of the Study Population

We received 1,078 completed questionnaires, leading to a response rate of 14.4%. The characteristics of the study population are presented in Table 1. The majority (57.3%) of the equine of the respondents were stated to be located in the cantons (states of Switzerland) of Bern, Zurich, Vaud, and Aargau (Supplementary Figure 2), matching with the spatial distribution of the equine population in Switzerland (40). The total number of equine owned by all respondents together resulted in 2,584 animals, with a median of two equine per respondent [range: 0–50, interquartile range (IQR): 1–3]. The median number of equine on the premises where the respondent's equine is stabled was 15 (range: 1–140, IQR: 5–26). The majority (55.5%, *n* = 598) of the equine owners transport their equine to other locations. The two most frequently selected reasons for transporting equine were attending a competition (61.7%, *n* = 369) and taking riding lessons (55.4%, *n* = 331).

#### Frequency of Observed Clinical Signs

All 1,078 respondents reported to have observed in total 17,016 clinical signs among their own equine and/or the equine on their premises during the last 12 months. The median number of clinical signs observed per respondent was 4 (range 0–340, IQR: 1–11). The most common observed clinical signs were pruritus (29.3%), respiratory signs (23.5%), lameness (19.1%), and diarrhea (14.5%) (Supplementary Figure 3). Overall, respondents contacted a veterinarian in 14.2% of the cases after observing clinical signs, with a median of 1 per respondent (range 0–60, IQR: 0–3) over the last 12 months.

#### MCA and Hierarchical Clustering of Factors Influencing Equine Owners' Perception and Attitude Toward CBS

The MCA and hierarchical clustering revealed three attitude clusters among the respondents with 41.4% (*n* = 446) of the respondents categorized to the highly positive attitude cluster, 52.2% (*n* = 563) to the moderately positive attitude cluster, and 6.4% (*n* = 69) to the negative attitude cluster (Supplementary Figures 4A–C).

The highly positive attitude cluster (*n* = 446) was characterized by the majority of the respondents within this cluster having highly positive attitudes toward factors mentioned in all sub-questions of question 21 and strongly agreeing to all statements of question 22. Among all reasons not to report, respondents from this cluster most frequently (52.0%) selected the answer “I don't have concerns.”

The moderately positive attitude cluster (*n* = 563) was characterized by the majority of the respondents having rather positive attitudes toward factors mentioned in all sub-questions of question 21 and rather agreeing to the statements of question 22. Among the reasons not to report, respondents of this cluster most frequently (42.0%) answered with concerns about privacy and data security.

The negative attitude cluster (*n* = 69) was characterized by the majority of the respondents having negative attitudes toward factors mentioned in all sub-questions of question 21 and strongly or rather disagreeing to the statements of question 22. In contrast, the majority within this cluster reported that “other reasons” (70.0%) was the main reason for non-reporting. General disinterest, the perception of reporting being highly time-consuming, and the perception that monitoring clinical signs is the responsibility of veterinarians were among the comments added as free text when selecting “other reasons” for not reporting.

#### Factors Influencing the Intentions Toward CBS

The majority (65.5%, *n* = 707) of the 1,078 respondents answered that they could certainly (12.5%, *n* = 135, “certainly yes”), presumably (26.4%, n = 285, “presumably yes”), or potentially (26.6%, n = 287, “maybe”) see themselves reporting clinical observations of their equine (question 18, outcome variable *would report*). Approximately a third (34.5,%, *n* = 371) of the 1,078 respondents answered that they would not (8.4%, *n* = 91) or rather not (26.0%, *n* = 280) report clinical signs they observed.

In the univariate logistic regression models, the variables *age, type of ownership, type of premises, profession, gender, language*, and *attitude cluster* were associated with a *p* < 0.2 with the outcome *would report*. The final multivariable logistic regression model indicated three significant independent variables, *language, attitude cluster*, and *profession* (Table 3). *French-speaking* compared with *German-speaking* respondents had an odds ratio of 2.31 (95% CI: 1.45–3.74) of being associated with the outcome of having positive intentions to use CBS and Equi-Commun. The odds of respondents belonging to the highly positive *attitude cluster* and negative *attitude cluster* were 11.29 (95% CI: 7.39–17.76) and 0.13 (95% CI: 0.05–0.30), respectively, in regard to their intention to use CBS compared with moderately positive *attitude cluster*. Furthermore, respondents with the *profession farmer* (OR: 0.36, 95% CI: 0.18–0.72), those with a profession related to the field of *animal health* (OR: 0.32, 95% CI: 0.12–0.84) or respondents who did not provide their profession (OR: 0.31, 95% CI: 0.15–0.64) reported to have significantly lower intentions to report clinical signs of their animals than respondents working in the human health field.

**Table 3:** Factors influencing equine owner's intentions to report to Equi-Commun, a community-based surveillance tool for equine health, resulting from a multivariable logistic regression analysis are presented.

**Factors**	**Levels**	***p*-Value**	**OR (95% CI)**
Language	German	–	Ref.
	French	<0.001	2.31 (1.45–3.74)
	Italian	0.095	10.19 (0.89–278.42)
Profession	Human health field	–	Ref.
	Working with equine	0.061	0.52 (0.26–1.02)
	Animal health field	0.022	0.32 (0.12–0.84)
	Farming	0.005	0.36 (0.18–0.72)
	Other profession	0.432	0.79 (0.43–1.42)
	I prefer not to say	0.002	0.31 (0.15–0.64)
Attitude cluster	Moderately positive attitude cluster	–	Ref.
	Highly positive attitude cluster	<0.001	11.29 (7.39–17.76)
	Negative attitude cluster	<0.001	0.13 (0.05–0.30)

### The CBS Tool Equi-Commun

Equi-Commun was technically functioning without issues after its launch on October 22, 2018. Until December 31, 2019, Equi-Commun received four reports by three unique users (Supplementary Table 3). These consisted of two cases of lameness, one case of colic, and one case of pastern dermatitis. None of the reports were explicitly related to infectious diseases or its suspect. None of the users registered to the system, instead they submitted their reports without registering. Because of its non-use, the Equi-Commun reporting tool website was inactivated at the end of December 2019.

### Qualitative Phone Interviews

Fifteen codes were identified during the analysis of the transcripts (Table 4). Among the 10 interviewed participants, all stated to have a positive attitude toward Equi-Commun. An example for quote for the code “Positive attitude toward Equi-Commun” was: “… when I read some of it, I thought, yes, that still sounds exciting, I think it's a good thing. When knowledge is acquired and the knowledge is later tried to be spread.”

**Table 4:** Codes created by intuitive coding using MAXQDA2020 Analytics Pro based on transcripts of semi-structured qualitative interviews among 10 equine owners regarding their perception of Equi-Commun (EC), definition of codes, and an example quote from the transcripts.

**Code name**	**Explenation of code theme**	**Quote examples from transcripts**
Positive attitude toward EC	Participant had a positive attitude toward EC	“…when I read some of it, I thought, yes, that still sounds exciting, I think it's a good thing. When knowledge, is acquired and the knowledge is later tried to be spread.”
Lack of memory	Participants could not or just partly remember the concept of EC	“Honestly, I know practically nothing about it [Equi-Commun].” “I don't remember it. It's a bit embarrassing because I really didn't know what Equi-Commun actually is. Yeah, no, I usually remember things like that, but obviously it didn't stick.”
Need of active information	Participants express their opinion for the need of more active information about EC	“I would do Facebook marketing with short, concise educational material written in the style of the equestrian revue or horse magazines. And I would do this seasonally on horse topics on things that are currently topics, now with the hay quality in autumn, with Cushing's [Cushing disease] or with worms etc.”
Found information through the internet	Participants got the information about EC through the internet by searching themselves or by coincidence	“I found this [Equi-Commun] on the Internet by accident.”
Suggestion for non-compliance: missing medical knowledge	Participants think that missing knowledge about equine in general and/or in the medical field is a reason for missing compliance	“Yes I think they [other equine owners] are afraid to report, or to report something wrong, or to interpret something that is wrong and that it is better that some professional does it.”
Suggestion for non-compliance: equine are healthy	Participants think that owners did not comply with the system because their equine were healthy	“So when I talk about me now, I have a horse that has no medical problem. Maybe they (persons who did not report) are all people who had extremely healthy horses.”
Suggestion for non-compliance: anxiety	Participants think that the anxiety of consequences due to notifying clinical signs might be a reason of missing compliance	“I believe that fears is there.” “And I also think there is fear that you could be convicted of something.” “Fear of being reported. It's quite possible that people will find it. Am I registered? Can I then perhaps no longer go and finish the (riding) course? And I always think it's something like that.”
Suggestion for non-compliance: lack of awareness about EC	Participants think that lack of awareness about EC among other equine owners could be a reason for missing compliance	“I might be able to tell you what happened to me. I filled out the survey once and then I kind of really forgot about it. I didn't realize anymore that something like this [Equi-Commun] existed and that you should do something about it.” “If then afterwards the horse has something that you probably don't even think about that you could/should report it… Yes, you might be a bit stressed afterwards and yes, your thoughts tend to be somewhere else.”
Did not understand the concept of CBS	Participants did not know the differences or the meaning of the terms clinical signs and diseases	Answer to the question if participant observed clinical signs after having explained the concept of EC: “For what disease again? Or in general?” “Yeah, the EHV-4, I could have reported it.”
Well informed	Participants found themselves well informed about EC	Answer to the question how the participant found the information provided about EC:“…but it was quite informative there.”
Limited interest	The interest of the participant in EC was limited	“I got that in a survey once, but I didn't follow it up.” “It simply hasn't had any relevance for me lately or hasn't become relevant yet. Now I have forgotten about it [Equi-Commun] ever since.”
Lacking information	The information provided about EC was perceived as lacking	“Because if you don't hear anything or have to search God knows where on the Internet until you can read up, I find it rather difficult.”
Doubts	Participants had doubts about the added value of EC	“But I then asked myself how developments can be mapped in a timely manner. So if you write something down or make an entry, is it simply statistically empirically afterwards or can you really use it directly and promptly? That was not so clear to me… That's why I'm not sure if it (Equi-Commun) will lead to a flood of information for what is expected to result as an output later.”
Good memory	Participant remembered EC well and was correctly informed about its aims	“I understand that Equi-Commun invites horse owners in particular to report any incidence of disease occurrences, especially those that are transmissible. And I have understood that Equinella is looking for this, especially from veterinarians.”
Misinformed	Participant was wrongly informed about EC	“So, I imagined that it is simply about the relationship between man and horse, what is good for the horses, what is bad for the horses. Something like that.”

Only few of the respondents mentioned limited interest in the tool. Some respondents stated that they previously felt well-informed about Equi-Commun, yet only few correctly remembered the aim and use of the CBS tool Equi-Commun. An example quote for the code “Misinformed” was: “So, I imagined that it (Equi-Commun) is simply about the relationship between man and horse, what is good for the horses, what is bad for the horses. Something like that.” This quote from one of the respondents points toward the lack of understanding that Equi-Commun was designed as a CBS tool to report clinical signs.

Several respondents mentioned to have gathered information about Equi-Commun over the internet and that they came across Equi-Commun randomly while searching for equine health content on the web. Some respondents further mentioned that they perceived active and repetitive information as necessary to improve compliance with the platform. To the question on what reasons other equine owners might have had for not reporting their observations to Equi-Commun, respondents mentioned the following ideas: (a) lack of awareness about Equi-Commun, (b) a possible anxiety of creating a negative impact if clinical signs were reported, (c) missing clinical knowledge among the equine owners regarding general issues about equine and medical understanding, and (d) that their equine were healthy, and thus they were not able to report health issues. An example quote for the code “Suggestion for non-compliance: lack of awareness about Equi-Commun” was: “I might be able to tell you what happened to me. I filled out the survey once and then I kind of really forgot about it. I didn't realize anymore that something like this (Equi-Commun) existed and that you should do something about it.”

## Discussion

The present study is the first attempt at determining the potential and challenges of CBS within the Swiss equine community. Additionally, this is the first study describing the process of establishing and disseminating a CBS tool for equine surveillance. Although the aim of Equi-Commun was to assess the benefit of surveillance data derived from a CBS approach compared with already existing equine health surveillance data, this aim was not achieved in the current project, as Equi-Commun received only four reports for the duration it was online.

The success of a CBS system is dependent on the perceived need of the community toward generating surveillance information. El Allaki et al. argued in their theoretical work on health surveillance theory that the initiation of a surveillance process requires three steps: (i) a dissatisfaction regarding the current (health) situation, (ii) a need for knowledge and/or time-dependent information, and (iii) some level of motivation to eliminate the dissatisfaction and to approach the information need on the population health status (41). Applying this concept to the equine CBS tool we have strived to implement, equine owners should have recognized and perceived a certain dissatisfaction regarding their equine's health and/or their surveillance in order to show compliance to a CBS approach. Or in other words, only if there was a strong enough perceived need for CBS in the Swiss equine community, such a system would have likely been successful. Indeed, the idea of Equi-Commun was created after stakeholders from the Swiss equine industry clearly stated their interest in being actively involved in the surveillance of equine health during a Swiss equine health network conference in 2016. Build on that, while our study has not directly assessed the dissatisfaction of equine owners nor the perceived need for CBS, it did assess the intentions of Swiss equine owners toward CBS by making use of an online survey. As it was found, the majority of the respondents (65.5%, *n* = 707) answered that they could see themselves reporting clinical signs. These aspects together could be regarded as a promising prerequisite for the success of a CBS approach, and we therefore expected to receive more interest in and reports submitted to Equi-Commun. However, this was not the case.

**Figure 1:**
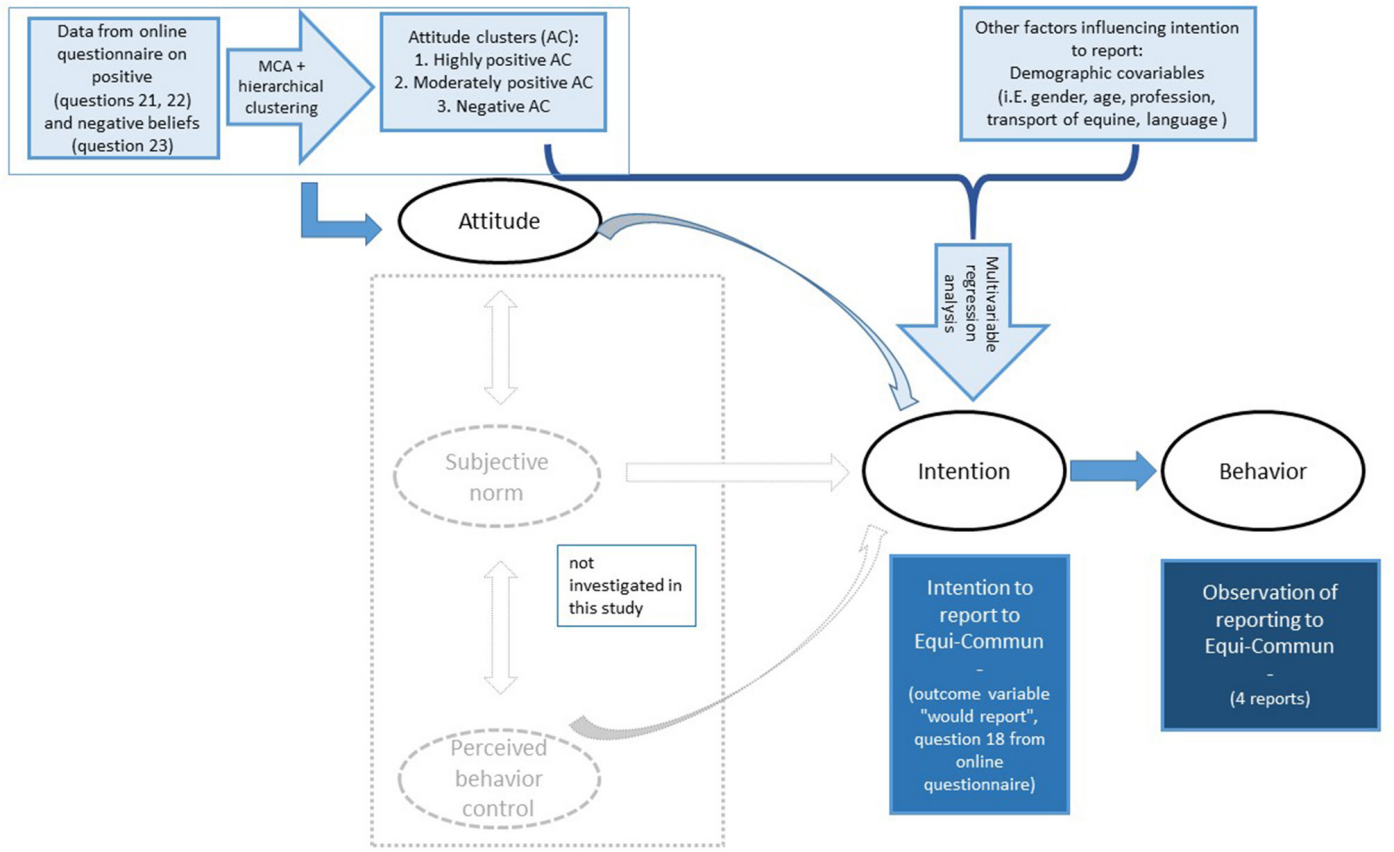
Framework of the Theory of Planned Behavior (TPB) by Ajzen (43) adapted to investigate factors influencing the intention and observed behavior to report to the CBS tool Equi-Commun.

Possible explanations for this non-use of Equi-Commun can be found in disciplines investigating the complexity of human behavior. According to the Theory of Planned Behavior (TPB), a specific human behavior related to a certain planned action is a product of humans' “intention” to carry out this planned action, their “attitudes” (i.e., values, priorities) toward the action, “social norms” (i.e., external expectations placed upon them), and their “perceived behavioral control” (i.e., their perceived ability to put actions of their choice into effect) in regard to the action (42, 43). In our study, we investigated the behavior of people, i.e., whether they used the CBS tool, by observing the reports submitted to Equi-Commun (Figure 1). On the other hand, we investigated the intention to report to Equi-Commun and the attitudes that may drive this intention through the online survey. The majority (65.5%) stated that they intend to report or at least maybe report to a tool, such as Equi-Commun. However, selection bias is expected to be prevalent for the online survey, as it is the case in most voluntary questionnaire-based studies (44, 45), which may have led to an overestimation of the equine community's rather positive intention. In particular, respondents have possibly reflected on the equine communities and their role in disease surveillance in a more positive way than they actually thought about it. The concept of responding to a possibly moral or ethical question in a way that an individual thinks the society expects them to respond is described in the so-called social desirability bias (44, 46). In addition, we assessed the attitude toward CBS and the intention to use it through the respondent's self-reflection on a description of the yet to be established Equi-Commun (Supplementary Material 1, section C, question 16). Hence, respondents were not able to reflect on previous experiences directly, yet only on their reflection on a hypothetical case scenario description of a CBS tool. Furthermore, in the qualitative telephone interviews conducted, after it was apparent that Equi-Commun would not be used, we again observed a positive perception toward the CBS tool, with all interviewees responding to clearly see a benefit in Equi-Commun. However, the selection bias in this group is even more expected, as participants of the telephone interviews were selected from the pool of online survey respondents that left their e-mail address voluntarily at the end of the questionnaire, in order to be updated in regard to Equi-Commun developments. In particular, later exploration of the qualitative interview participants after conducting the MCA revealed that 4 of 10 interviewees were assigned to the highly positive attitude cluster, whereas 6 were assigned to the rather positive attitude cluster. No interview was conducted with persons from the negative attitude cluster. Thus, saturation of perspectives among equine owners with different attitudes toward CBS could not be reached. It is likely that telephone interviewees, similar to the respondents of the online questionnaire, represent the more interested equine owners with positive attitudes toward CBS, than average.

We investigated the factors collected during the questionnaire as drivers of the observed intention of respondents to submit reports to Equi-Commun. Equine owners categorized in the highly positive attitude cluster were nearly 12 times more likely to have a positive intention toward reporting to Equi-Commun than individuals from the moderately positive attitude cluster. Interestingly, the respondents belonging to the respective attitude cluster answered to all sub-questions in a similar way, suggesting that they either fully support a system, such as Equi-Commun (highly positive attitude cluster), or deny it (negative attitude cluster), whereas the moderately positive attitude cluster is somewhere in the middle. This might indicate that type of incentives for reporting clinical signs is not crucial (e.g., by receiving information material, profit from the own equine health diary, getting feedback from the system about the health status in the Swiss or regional equine population)—either persons have a positive attitude, therefore see certain incentives and would like to profit from all benefits, or they have a negative attitude toward the tool, therefore prefer not to report. The same picture was apparent in regard to the perception on how CBS would help to improve the health of the own equines, the equines from the premises, the equines in the region, or all equines in Switzerland. Again, the respondent supports either most of these statements or almost none. This clear separation of the respondents leaves little room for motivating the respondents seeing no benefit at all (individuals from the negative attitude cluster)—fortunately, this cluster consists of only 6.4% of the population.

We have also revealed that respondents who classified themselves as farmers and those working in the animal health field (including veterinarians) are less likely to report to a CBS tool, such as Equi-Commun, than equine owners working in the human health field. This could be due to the negative perception of these professional groups toward collecting health data in addition to the currently mandatory data documenting needs (42, 47). In light of existing surveillance, monitoring, control programs, and respective documentation responsibilities, the burden for committing to an additional surveillance system, such as a CBS tool, is possibly higher for equine owners who are part of the farming and animal health sectors than for equine owners of other professions. Crosslinking of existing health data might therefore be of upmost importance to disburden professional animal owners from data reporting. On the other hand, engaging equine owners from these health fields might bring more potential for a CBS tool, because they are expected to be able to deliver better quality and higher quantity data than other professions owning few animals.

Finally, we observed that the odds were 2.3 times higher for the intention to report to Equi-Commun in French-speaking equine owners than in German-speaking owners. Such differences in attitudes along language borders within Switzerland have already been reported in different fields. For example, acceptance of replacing nuclear power plants was lower among French-speaking Swiss individuals than among German-speaking (48); or the agreement of Swiss physicians toward end-of-life decisions by use of lethal drugs was higher among French-speaking than among German- and Italian-speaking medicals (49). Such differences based on the language-use and language regions are likely due to cultural differences, acceptance of certain habits, and overall positioning in regard to health and health-related topics. Targeted project communications and information dissemination should therefore take them into account.

Within our study, we were able not only to investigate the attitudes and intention of the equine owners in regard to CBS but also to test their actual behavior. The actual behavior of interest in this contexts is defined as the reporting of clinical signs to Equi-Commun. With the majority of the respondents having stated positive intention toward CBS and only four reports recorded in Equi-Commun, we clearly observed a so-called intention-behavior gap. This concept describes the discrepancies between human intention to perform a certain behavior and them acting accordingly and has found particular interest in the research of medical and lifestyle behavior of patients within the health sector (50–52). Evidence shows that intentions get translated into actions in only about half of all cases (51). Obstacles influencing the intention-behavior gap can be divided into three main categories: getting a new tool started (e.g., in our case, setting the intention to report clinical signs), keeping it ongoing (e.g., keeping informed about Equi-Commun), and reach the goal (e.g., the actual act of reporting to Equi-Commun) (51). Possible explanations for the observed intention-behavioral gap may be found in each of these three obstacles. However, even though critically important, elaborating the complexity of equine owners' intentions and how they translate it into behavior have to be deferred to a next study investigating these concepts and how these apply to community-based animal health surveillance.

Engaging stakeholders in CBS is complex and requires their active involvement starting by assessing the need toward CBS as well as throughout the implementation process. In their conceptual study on fish farmers' potential in aquatic syndromic surveillance, Brugere et al. emphasized that the authority of veterinarians and diagnostic laboratories must be extended to include farmers (53). According to the authors, farmers should be acknowledged as the starting point of disease surveillance, with equal power and responsibility. Therefore, including relevant stakeholder's knowledge, opinions, and needs as well as methods and tools to ensure such inclusive processes must be guaranteed for a successful CBS, already during its conceptualization. Within our study although we attempted to investigate the wide equine community's attitude toward a CBS tool through the online questionnaire, members of the community were not included in the development of the tool. We would have possibly been more active in uncovering the underlying dynamics in equine owner's surveillance behavior holistically and continuously by applying transdisciplinary approaches to co-constructing CBS in the Swiss equine community. As an example, in the beginning phase of the project, regular stakeholder workshops with equine owners could have been organized to start assessing the overall attitude toward CBS and to better assess its needs in this community. Such workshops should be accompanied by experts from social sciences, such as sociology, psychology, and anthropology (54, 55). Intervention mapping, a theory- and evidence-based framework providing a systematic and stepwise approach toward planning health interventions, may have been other concepts and tools worth to be consulted for planning, developing, and implementing a CBS system (56). Intervention mapping is grounded in community-based participatory research methods to ensure that the intervention matches priority population needs, and thus may have been a useful tool to investigate equine owners' underlying thought processes and dynamics in regard to CBS.

One of the most relevant shortcomings of successfully implementing a CBS system may probably lie in project dissemination, communication, and marketing. An effective CBS approach requires personal staff dedicated to manage the project, continuously contact, inform and support community members in collecting data, maintain a database, analyze and visualize data, and disseminate analysis outcomes (57). Within the scope of our study, we have adapted several strategies to disseminate Equi-Commun effectively (Supplementary Table 2). The qualitative interviews, however, revealed that even though equine owners felt well-informed, most of them could not remember Equi-Commun and its objectives correctly. This suggests that effective project communication has failed. In a study in northern Australia and Papua New Guinea, researchers investigated factors influencing the acceptability and value of CBS for dog rabies (58). The authors revealed that verbal communication, such as direct conversations, radio, and community meetings, was mentioned the strongest, whereas social media posts (depending on the region and age of community members) and print media were less likely to be valued by community members. A study conducted among Swedish dairy cattle farmers suggests that consistent, persistent, audience-tailored, benefit-revealing, and personal contact and communication between receivers and providers of data are key assets to a successful and continuous data collection (12, 20). Potential action points included oral and participatory information exchange during data collection, refresher training workshops for community-based animal health workers, or rural radio programs with disease information spread for cattle farmers (20). These experiences show how specific feedback operations must be to meet the exact needs of data providers in order to maintain compliance. In our study, we revealed that although information provided by the project management team during the communication phases was perceived as clear and understandable, it was not efficient and persistent enough to be remembered after 1 years' time. This demands for consistent, targeted and more frequent information campaigns. Profound trans- and interdisciplinary approaches for project communication and dissemination through the inclusion of equine owners and experts from social sciences, in addition to the veterinary epidemiologist and equine practitioner, could have substantially benefited the implementation of Equi-Commun. Researchers and surveillance practitioners planning to translate their CBS ideas to practice should make use of existing methodological frameworks and toolkits from the implementation science field, which encompasses the right tools for narrowing the gap between implementation in research settings and implementations of programs intended to be used in everyday practices (59). These have been approaches and methodologies not made use of during the implementation of Equi-Commun. However, when planning implementation strategies and more resource demanding interventions for setting up a CBS system, the benefit should be weighed in comparison with the necessary resources, such as personal, finances, and time. Even though CBS systems can be less material demanding than active surveillance system (e.g., continuous serological surveillance), certain “hidden” resources needed to set up and maintain the system have to be accounted for. In the case of CBS within the equine community in Switzerland, despite the given interest and potential of equine owners to observe clinical signs, the benefits of having CBS data as additional surveillance information would not have overweigh the efforts and resources required.

Furthermore, the relatively high level of equine health among the Swiss population was a potential reason for the equine owners' non-compliance to Equi-Commun. This was confirmed by interviewees of the qualitative survey mentioning their equine's good health as a reason for non-reporting of clinical signs. Although census studies on the health or diseases of the Swiss equine population are lacking, judging by the low number of official reports on notifiable infectious diseases–which encompassed only five cases of Salmonellosis and one case of Contagious Equine Metritis (CEM) within 1 year (07.14.2018–07.14.2019)–support the argument that at least critical equine infectious diseases are rare (60). Similarly, although a voluntary reporting system of non-notifiable diseases, and therefore not expected to be thoroughly representative of each disease event in the equine population, reports submitted to Equinella have also been rather low in number (34). On the other hand, respondents of the online questionnaire reported as a median to have observed four times clinical signs among their equine within a year. Additionally, respondents of the online questionnaire stated to have contacted a veterinarian in only 14.2% of all observed clinical cases. This is pointing toward that the information of a great majority of clinical signs observed by equine owners does not get forwarded to veterinarians in the first place. Therefore, in case Equi-Commun would be more present in the equine owners' mind, there is potential for reports in a CBS tool. It is noteworthy that the large majority of clinical signs observed (mostly pruritus, lameness, and respiratory signs) are not clearly related to infectious diseases. This suggests that while clinical signs of infectious diseases might be rather rarely observed, such related to non-infectious diseases may be used as a motivation of equine owners to record their animal's health diary, and as such promote CBS tools also for infectious disease. Nonetheless, the currently known good health status of equine in Switzerland does not urge the requirement of a CBS system as an addition to existing surveillance systems, particularly in terms of covering further infectious disease surveillance.

## Conclusion

This study contributed to the little explored potential in equine owner's observations of clinical signs used for continuous surveillance of equine diseases, by assessing the equine owners' attitudes and intentions toward CBS and by developing and testing a CBS tool, named Equi-Commun. The intention of contributing to disease surveillance among equine owners is given, and equine owners detect health issues of their animals on average four times per year. However, we observed a clear intention-behavior gap, as the implemented CBS tool was not used among the equine owners. We here identified three critical, interlinked issues that may have led to the non-use of Equi-Commun within the Swiss equine community: (1) the need for surveillance within the community of interest must be given and should be assessed before implementing CBS; (2) the respective population under surveillance, here the equine, needs to show enough relevant clinical cases for equine owners to be able to maintain the memory of an existing tool and its possible use, and (3) targeted and high effort communication and management of the system is key for its success. While CBS relying only on lay animal owners could potentially provide a good proxy of timely surveillance data, complementary to existing surveillance systems, it is questionable whether the added value of generated surveillance knowledge is in balance with the efforts necessary to implement a successful system. With this study, we showcased both the potential and challenges of CBS in animal health, as this may be of relevance and guidance for similar future initiatives.

## Data Availability Statement

The raw data supporting the conclusions of this article will be made available by the authors, without undue reservation.

## Ethics Statement

Ethical review and approval was not required for the study on human participants in accordance with the local legislation and institutional requirements. The participants provided their written informed consent to participate in this study.

## Author Contributions

RÖ designed and conducted the online questionnaire, co-worked on the conceptualization and implementation of Equi-Commun, conducted the information dissemination and management of Equi-Commun, supervised the qualitative interviews, conducted data analysis and wrote the first draft of the manuscript. SK conducted the qualitative interviews and applied qualitative analysis on them, as well as wrote parts of the manuscript. VV has helped to conceptualize and reviewed the online survey and qualitative interview questions, as well as helped to analyze the data. DH and FR-W have contributed to the conceptualization of Equi-Commun and the online survey. SD has designed the study and supervised all parts. All authors contributed to the data interpretation, manuscript revision, read, and approved the submitted version.

## Conflict of Interest

The authors declare that the research was conducted in the absence of any commercial or financial relationships that could be construed as a potential conflict of interest.
