# Supplementary material for: Potential and Challenges of Community-Based Surveillance in Animal Health: A Pilot Study Among Equine Owners in Switzerland
Source: Front Vet Sci. 2021 Jun 4;8:641448. doi: 10.3389/fvets.2021.641448 (PMC8212947; doi:10.3389/fvets.2021.641448)
Supplement: Supplementary file 1 [file Data_Sheet_1.pdf]

Supplementary Material
for the article
"Potential and Challenges of Community-Based Surveillance in Animal Health: A Pilot Study Among Euine Owners in Switzerland"

1 Supplementary material

Supplementary material 1. Online questionnaire, translated from its original language (German) to English for the use of this publication. Questions marked with "*" were mandatory to answer.

"Equi-Commun" – the online-reporting platform for disease signs from equine in Switzerland

Dear equine owners,

Dear equine keepers,

Thank you for being interested in this survey!

With this survey we want to investigate your interest in the reporting of clinical signs you observe in your equine to the online platform **Equi-Commun**. This will give us an insight if a reporting platform for clinical signs of equine living in Switzerland would be successful. You will get more information about **Equi-Commun** in this survey. Based on the outcomes of this survey we will have the chance to adjust the platform accordingly.

If you have any questions about this survey, its outcomes or our research please contact Ranya Özcelik (ranya.oezcelik-at-vetsuisse.unibe.ch).

Thank you for your participation!

The Equi-Commun Research Team of VPHI

In this survey all animals of the genus equine, such as horses, ponies, donkeys, hinnies and mules, are pooled as **"equine"**. Please answer the questions equally, regardless of which equine type you own or keep.

We would like to inform you about the following:

- The survey will last about 15 to 20 minutes.
- At the end you will be asked some personal questions.
- If ever possible, please answer all the questions. A gapless recording of your answers is very significant for our analysis.
- Please answer the questions sequentially.
- You have to be at least 16 years old.

- The data gained will be used for scientific research at University Bern.
- Your answers will be stored anonymously.

A. QUESTIONS ABOUT THE EQUINE YOU OWN AND/OR KEEP

1 Are you…?*

Please only choose one of the following answers:

- ☐ Equine keeper
- ☐ Equine owner
- ☐ Both
- ☐ Other (please define in the comment box)
    - o Please write a comment about your choice.

2 How many equine do you own?*
Only answer this question if following requirements are given:
Answer was "equine owner" or "both" or "other" (please define in the comment box) at question number 1 (Are you…?)
In this box you only must fill in numbers.
Please put your answer here:

3 How many equine are there on total on the premise your equine is habited?*
Only answer this question if following requirements are given:
Answer was "equine owner" at question number 1 (Are you…?)
In this box you only must fill in numbers.
Please put your answer here:
If you don't know the exact number, do an estimate.

4 How many equine do you keep on your premise (own equine and equine from others)?*
Only answer this question if following requirements are given:
Answer was "equine owner" or "both" or "other" (please define in the comment box) at question number 1 (Are you…?)
In this box you only must fill in numbers.
Please put your answer here:
If you don't know the exact number, do an estimate.

5 What type of premise do you own or what type of premise does your equine habitat on?*
Please only choose one of the following answers:

- ☐ Agricultural premise with equine (boarding and/or own equine) and other farm animals

- ☐ Stable for boarding equine (not agricultural)
- ☐ Private equine stables
- ☐ Other (please describe)
- ☐ I don’t know

Please write a comment about your choice.

6 How is the living space/stable of your equine?

Please choose all the right answers:

- o Box stalls (single equine)
- o Box stalls with direct access to outdoor free range (single equine)
- o Box stalls with daily outdoor free range on pasture (single equine)
- o Individual box stalls with direct access to outdoor free range in a group
- o Group housing in a free range stable
- o Single equine on pasture full time
- o Group of equine on pasture full time
- o Other:

7 On how many different locations are your equine kept at?

Please only choose one of the following answers:

- ☐ 1
- ☐ 2
- ☐ 3
- ☐ More than 3

Different places means locally separated premises, stables or lodgings.

8 In which canton/s is/are this/these locations?

Please choose all the right answers:

- o Aargau
- o Appenzell Innerrhoden
- o Appenzell Ausserrhoden
- o Basel-Landschaft
- o Basel-Stadt
- o Freiburg
- o Genf
- o Glarus
- o Graubünden

- Jura
- Luzern
- Neuenburg
- Nidwalden
- Obwalden
- St. Gallen
- Schaffhausen
- Solothurn
- Schwyz
- Thurgau
- Tessin
- Uri
- Waadt
- Wallis
- Zug
- Zürich
- Other:

9 How often do you visit your equine?

Please only choose one of the following answers:

- I am always present
- Several times a day
- Once a day
- Once a week
- Several times a week
- Once a month
- Less

10 Are your equine sometimes transported (for excursions, trips) to other places?*

Please only choose one of the following answers:

- Yes
- No

11 What are the reasons for your excursions or trips with your equine?*

Only answer this question if following requirements are given:
Answer was "yes" at question number 10 [transport] (Are your equine sometimes carried to other places?)

Please choose all the right answers:

- Ride-out on a distant terrain
- Visiting a veterinary practice or an animal hospital
- Tournaments
- Riding lessons
- Breeding shows
- Equine markets
- Holyday camps
- Other:

12 On these places, is your equine in contact with other equine?*

Only answer this question if following requirements are given:
Answer was “yes” at question number 10 [transport] (Are your equine sometimes carried to other places?) Please choose the right answer for every point.

Contact

- ☐ Never
- ☐ Rarely
- ☐ Sometimes
- ☐ Often
- ☐ Always

Contact: for example sniffing each other, equine held together jointly, sharing of water dispenser and feeding places.

B. CLINICAL SIGNS OBSERVED

In this part of the survey we would like to ask you about your perception and awareness regarding disease signs (signs) of your equine or the equine in your stables.

13 Following you will find a list of signs you once might have observed on your equine or the equine standing in your stables.

How many times did they show up <u>during the last year</u>? *

Note: ***If an equine shows the same clinical signs over several continuous days, consider as one case/ one time.***

In this question box you only must fill in numbers.

Every answer has to be between 0 and 100.

Frequency of the observed clinical sign

- Weight loss
- Abortion (premature departure of the embryo)
- Stillbirth (foal that dies shortly before or during the birth after a normal gestation period)
- Malformation of the foal (inherent)
- Pale mucous membrane (almost white mucous membrane)
- Diarrhea
- Fever
- Itching
- Respiratory signs (for example: cough, nasal discharge, forced and/or increased breathing, respiratory sounds)
- Neurologic signs (for example: drowsiness, uncoordinated walk, abnormal behavior, misalignment)
- Lameness
- Death of unknown cause
- Death of known cause

14 What other clinical sign have you observed?

Please fill in your answer here:___________ .

15 How often did you contact a veterinarian because of clinical signs of an equine?

In this box you only must fill in numbers.

Every answer must be at least 0.

Frequency of contacting the veterinarian during the last year: _______ .

C. QUESTIONS ABOUT "EQUI-COMMUN" AND MOTIVATION ABOUT PARTICIPATION

Please read the following text attentively:

“Equi-Commun” was developed as a part of a research project at Veterinary Public Health Institute (VPHI) of the University of Bern. It addresses all interested equine owners and equine keepers in Switzerland. On the online reporting tool **Equi-Commun** all of your equine’s clinical signs can be reported voluntarily via computer, tablet or smartphone.

An example: You realize that your equine has been coughing increasingly over the last two days. You go the website of the reporting platform **Equi-Commun** and report the clinical sign you observed. You further will be asked to give some specification about your equine (name and age) and the length of your observation (when did you first observe this clinical sign and how long have you been observing it). If you contacted a veterinarian, you can also state this. After finishing, the report will be transmitted to our research group at the VPHI. Registered equine owners/ keepers will be able to see their reports in an user-only internal area of the platform. The VPHI research team of **Equi-Commun** will have the overview over all incoming reports. These will then be observed and analyzed centrally by the research team. Thus, the research team is allowed to have a good insight about currently existing clinical signs, which could indicate a disease event or an epidemic within the equine population. However, these reports cannot replace any veterinary opinion or possible consultation.

16 Do you see a benefit in the idea of the online-reporting platform Equi-Commun? *

Please only choose one of the following answers:

- ☐ No
- ☐ Rather no
- ☐ I am uncertain
- ☐ Rather yes
- ☐ Yes

17 Which benefits do you see in such an online-reporting tool?

Only answer this question if following requirements are given:
Answer was “Rather yes” or “I can´t judge it” or “Yes” at question number 16 [benefit] (Do you see a benefit in the idea of the online-reporting platform “Equi-Commun”?)
Please put your answer here:_________________________ .

18 Can you see yourself reporting your observations to a centralized reporting platform (such as Equi-Commu)? *

Please only choose one of the following answers:

- ☐ Not at all
- ☐ Probably not
- ☐ Maybe
- ☐ Presumably yes
- ☐ Certainly yes

To report in this context means: to feed in in a centralized platform clinical signs you observed among your equine. You as a reporting person have (after a voluntary registration) access to the data you reported. Third parties won’t be able to see them. The reports will be treated confidentially and will only be seen by the research team.

19 Which of the following signs you would collect within “Equi-Commun”? *

Only answer this question if following requirements are given:
Answer was “Probably not” or “Maybe” or “Presumably yes” or “Certainly yes” or “Other” at question number 18 [system acquisition] (Can you imagine to report on a centralized reporting platform (such as “Equi-Commun”) equine’ signs that you observed?)

Please choose all the right answers:

- All
- Miscarriage (premature departure of the embryo)
- Weight loss
- Stillbirth (foal that dies shortly before or during the birth after a normal gestation period)
- Malformation of the foal (inherent)
- Pale mucous membrane (almost white mucous membrane)
- Diarrhea
- Fever
- Itching
- Respiratory signs (for example: cough, nasal discharge, forced and/or increased breathing, respiratory sounds)
- Neurologic signs (for example: drowsiness, uncoordinated walk, abnormal behavior, misalignment)
- Lameness
- death of unknown cause
- death of known cause

20 If there are clinical signs you would not report, what are your reasons for that? *

Only answer this question if following requirements are given:
Answer was at question number 19 [system acquisition] (Which of the following signs you would collect within “Equi-Commun”?)

Please choose all the right answers:

- I don't dare to recognize some signs.
- I don’t know some signs.
- I don’t want to get my premise in trouble.
- I believe some signs are not worth to be reported.

- I am concerned about privacy.
- Other

21 Which of the following statements motivate you to report clinical signs of your equine into an online reporting platform like "Equi-Commun"? *

Please choose the right answer for every point.

Individual reasons:

- I care about early detection of infectious diseases in my premise because of financial reasons.
- My reports are listed continuously on "Equi-Commun". Therefore I get an overview about the reported health issues of the equine.
- Receiving regularly newsletter with current information about "Equi-Commun" and about the current situation of equine diseases in Switzerland and foreign countries.
- The insight to a map of Switzerland on which all registered reports are (anonymously) illustrated.
- Receiving an info-email in case of a striking increase of a sign in my region.
- Receiving an info-SMS in case of a striking increase of a sign in my region.

Likert-scale answers:

- Doesn't motivate me at all
- Doesn't motivate me that much
- I don't care
- Slightly motivates me
- Motivates me a lot
- Doesn't apply to me

22 How do you feel about the following statements?

"By observing and reporting signs I support early detection of infectious diseases, …*

Please choose the right answer for every point.

- …and therefore protect the health of my equine.
- …and therefore protect the health of all equine on the premise.
- …and therefore protect the health of all equine in the region.
- …and therefore protect all equine in Switzerland.

Likert-scale answers:

- I don't agree

- I rather don’t agree
- I don’t care
- I rather agree
- I agree completely

23 What would be reasons for you to not report on Equi-Commun? *

Please choose all the right answers:

- Concern about privacy (address, email address, etc.)
- Concern to report a wrong observation
- Concern about a bad reputation of my premise
- I don't have concerns
- Other concerns

24 What of the additional following information do you agree do register on “Equi-Commun”?

Please choose all the right answers:

- Canton of the stable/premise
- Postal code of the stable/premise
- Name of the stable/premise
- Your relation to the equine (are you equine owner, equine keeper, both or something else)
- Information about the equine (age, gender, breed, use)
- Number of equine in the premise/ on the farm
- Name or office of your veterinarian

25 Which technical device would you prefer to register the sign-report?

Please only choose one of the following answers:

- An application on smartphone or tablet
- On an internet browser on a computer and/or laptop

D. DEMOGRAPHIC QUESTIONS

26 In which occupational field do you work?
Please only choose one of the following answers:

- Full time with equine
- Part time with equine

- In human health
- In animal health
- Agriculture
- I don’t want to tell
- Other

27 What is your birth year? *

Your answer has to be between 1918 and 2002.

In this field only fill an integer number.

Please fill in your answer here:_________ .

28 What is your gender? *

Please only choose one of the following answers:

- ☐ Woman
- ☐ Man
- ☐ I don’t want to tell

29 What is the postal code of your place of residence? *

In this field only must be numbers.

Please fill in your answer here:_______________ .

30 Are you interested in the results of this survey?

If so, please fill in here your email-address.

Your email-address will be saved separately from the rest of your answers. We will not be able to conclude with your answers.

Please fill in your answer here:_______ .

31 Do you have any comments about the survey?

Please fill in your answer here: ______ .

2 Supplementary figures

A

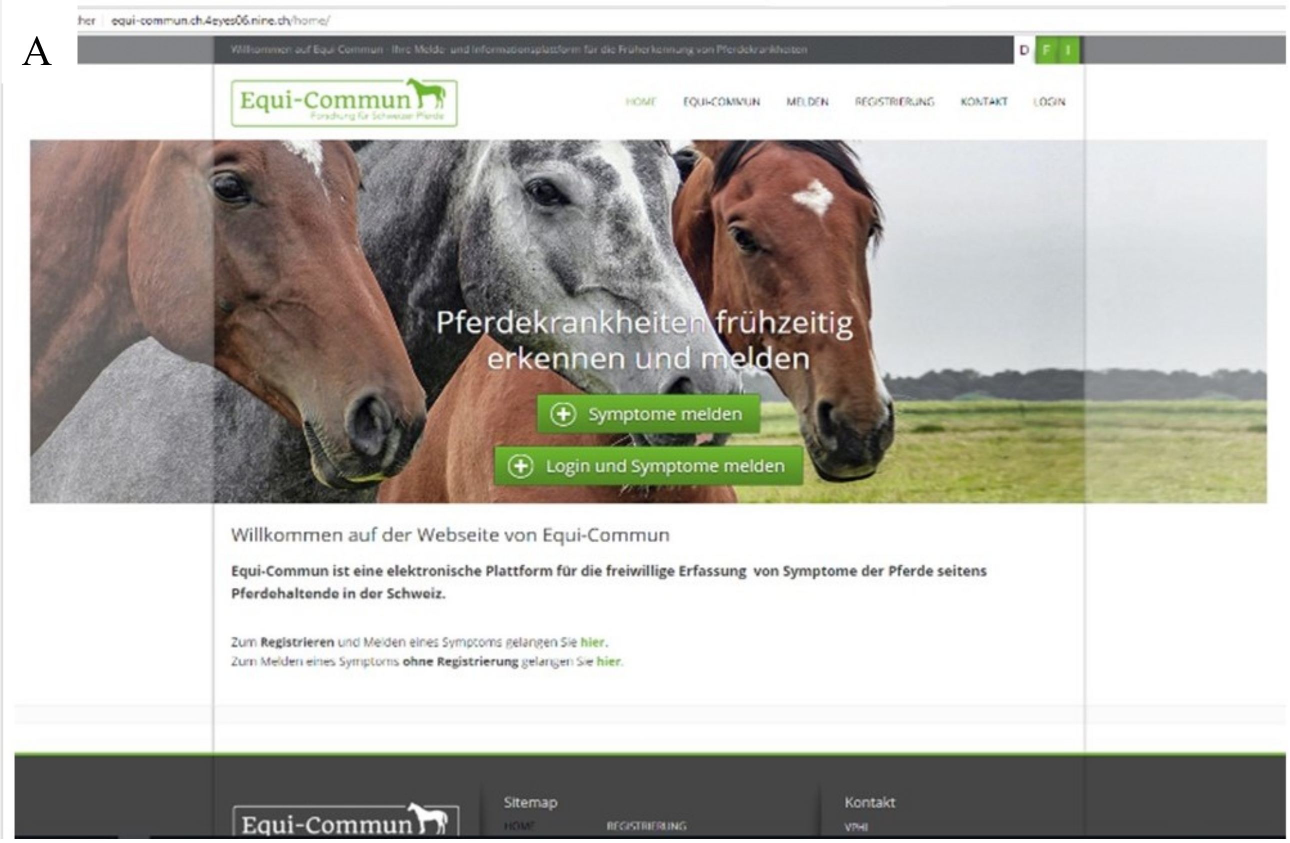

B

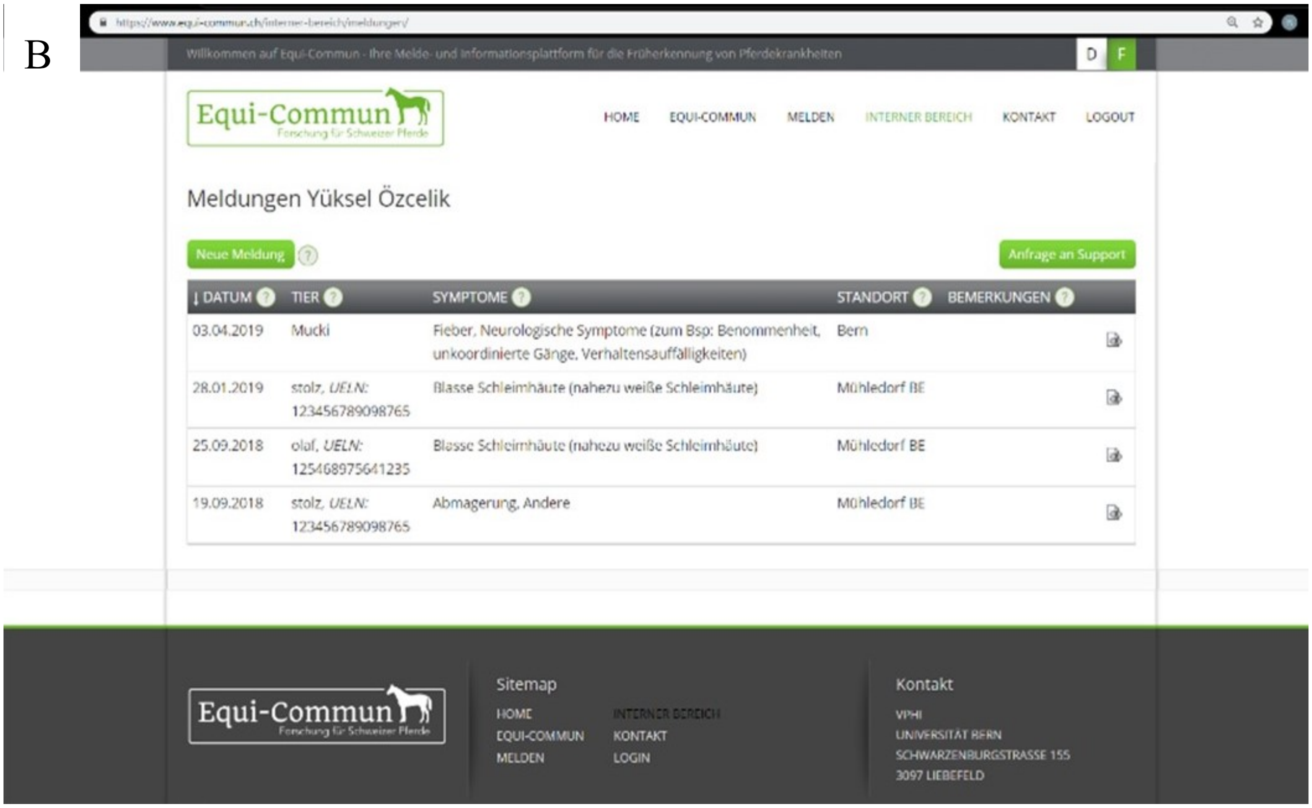

Supplementary figure 1 (A) and 1 (B). (A) Screen-print of the Equi-Commun homepage. (B) Screen-print of the user interface of Equi-Commun. List of submitted reports (dummy data) of one mock-user in the internal space.

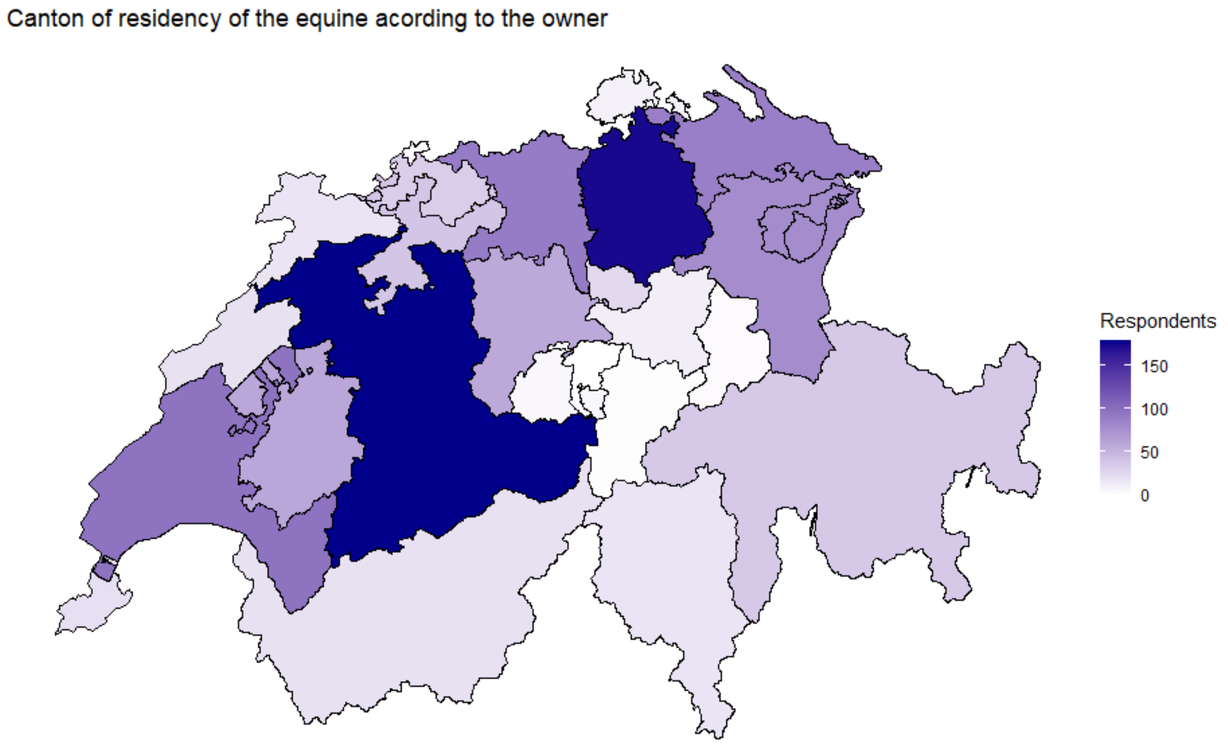

Supplementary figure 2. Distribution of the equine by cantons (member states of the Swiss confederation), according to the information provided by the equine owner in the online questionnaire.

A

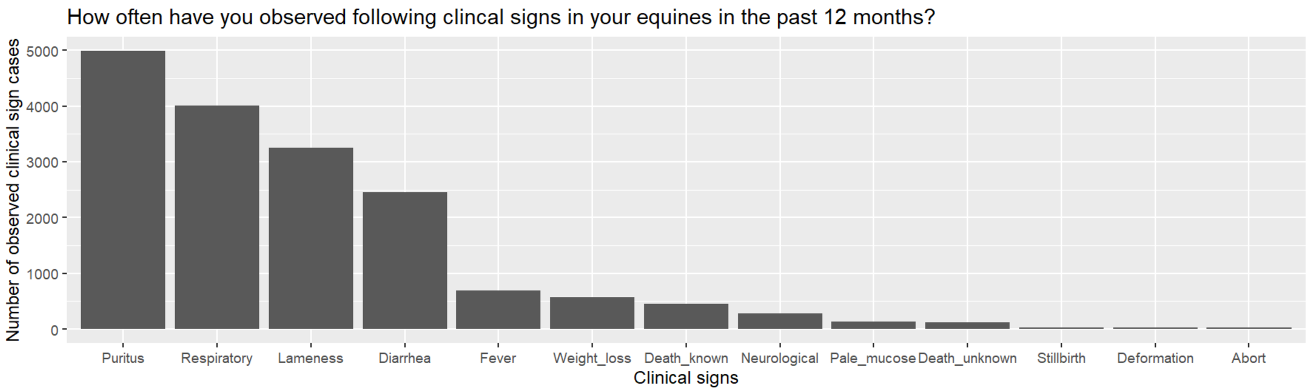

B

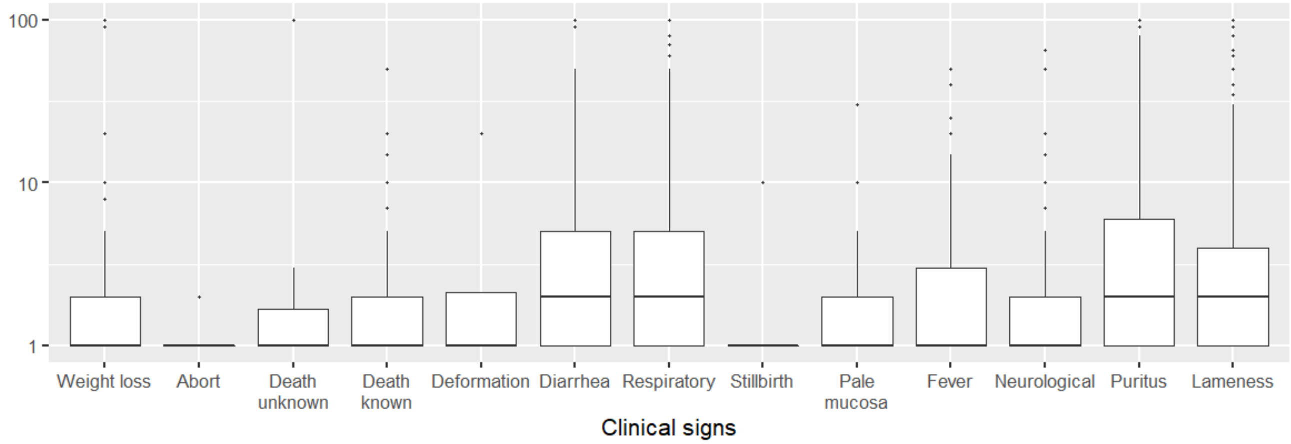

Supplementary figure 3 (A) and 3 (B). (A) Number of clinical signs observed by respondents of the online survey amongst their equine in the past 12 months. **(B)** Median and percentile of the number of clinical signs observed per equine owner amongst their equine within the past 12 months, separated by clinical sign category. The y-axis was log transformed.

A

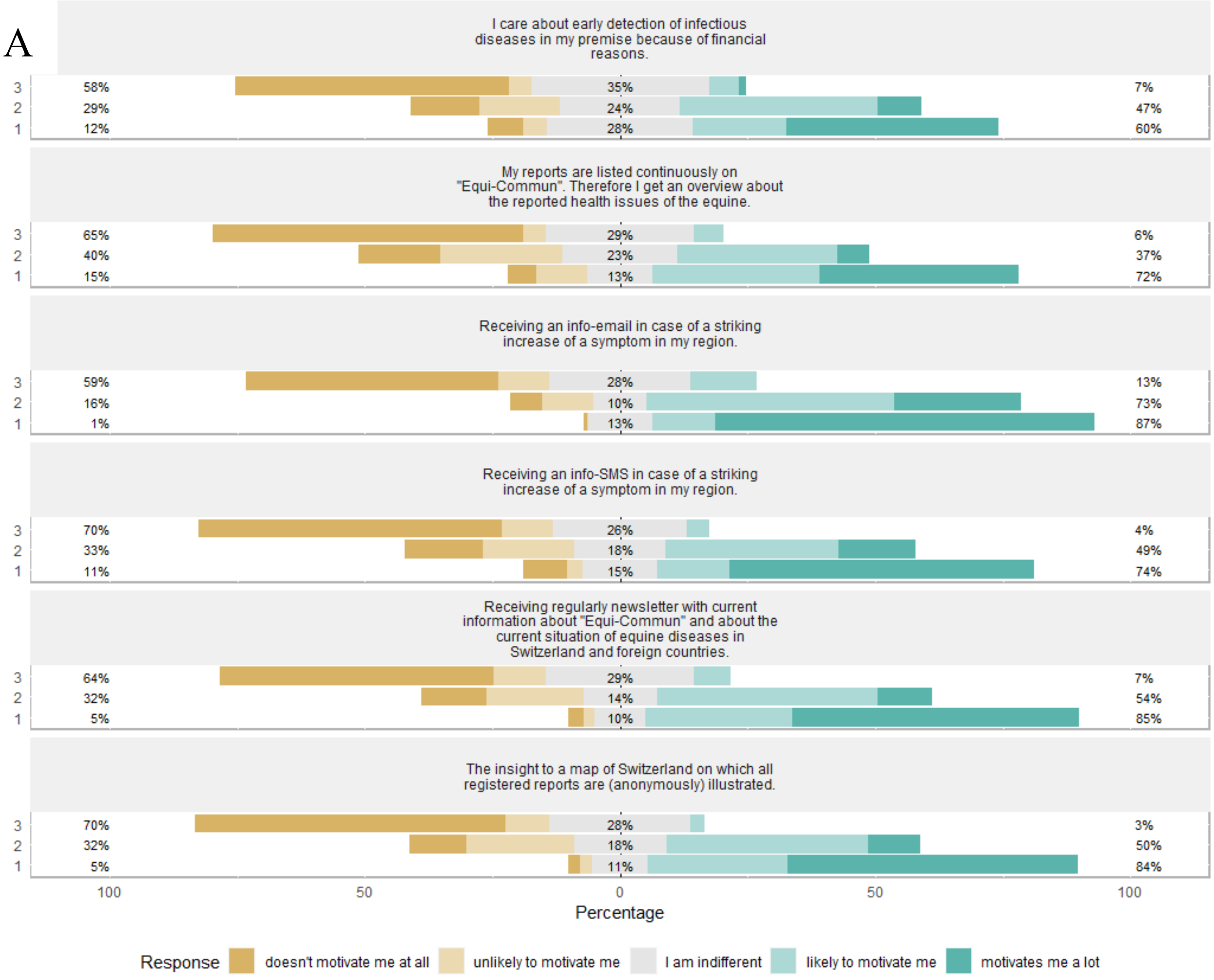
I care about early detection of infectious diseases in my premise because of financial reasons.
My reports are listed continuously on "Equi-Commun". Therefore I get an overview about the reported health issues of the equine.
Receiving an info-email in case of a striking increase of a symptom in my region.
Receiving an info-SMS in case of a striking increase of a symptom in my region.
Receiving regularly newsletter with current information about "Equi-Commun" and about the current situation of equine diseases in Switzerland and foreign countries.
The insight to a map of Switzerland on which all registered reports are (anonymously) illustrated.
Percentage
Response
doesn't motivate me at all
unlikely to motivate me
I am indifferent
likely to motivate me
motivates me a lot

B

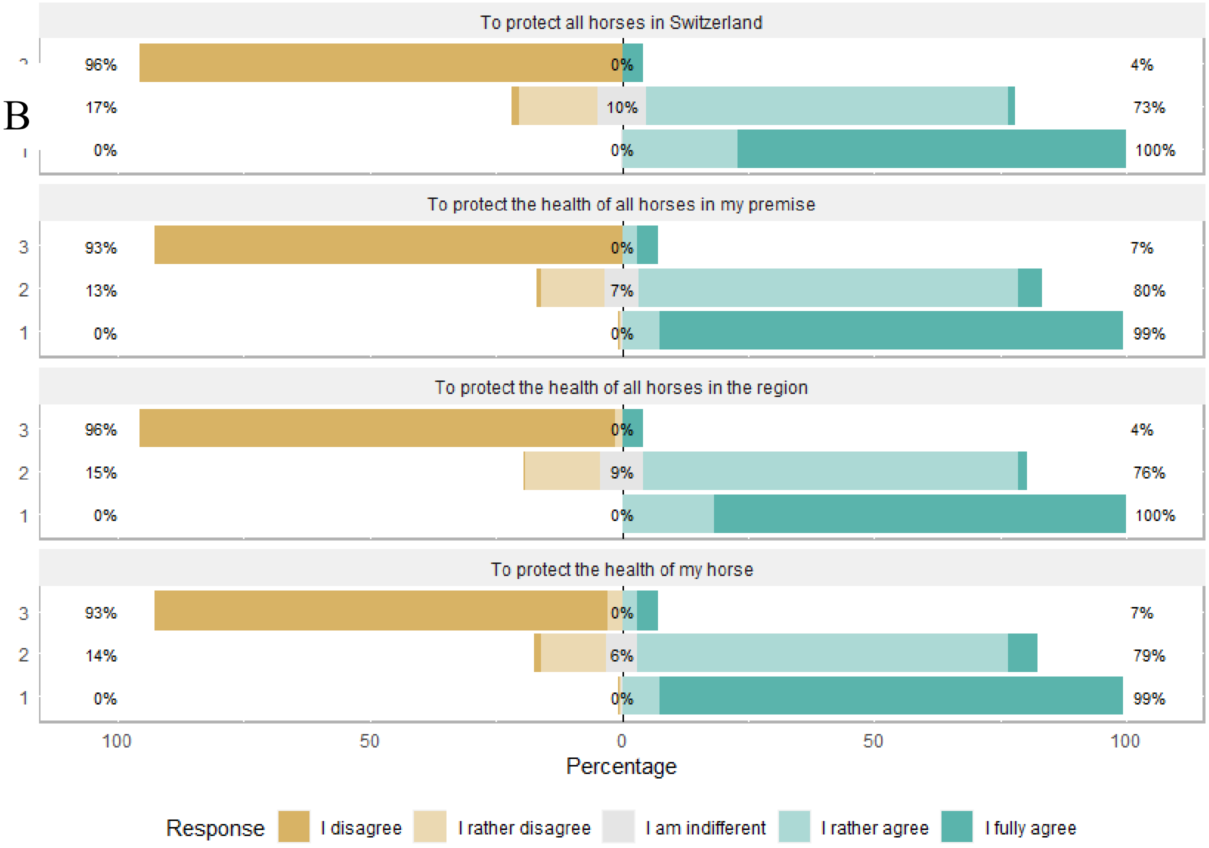
To protect all horses in Switzerland
To protect the health of all horses in my premise
To protect the health of all horses in the region
To protect the health of my horse
Percentage
Response
I disagree
I rather disagree
I am indifferent
I rather agree
I fully agree

C

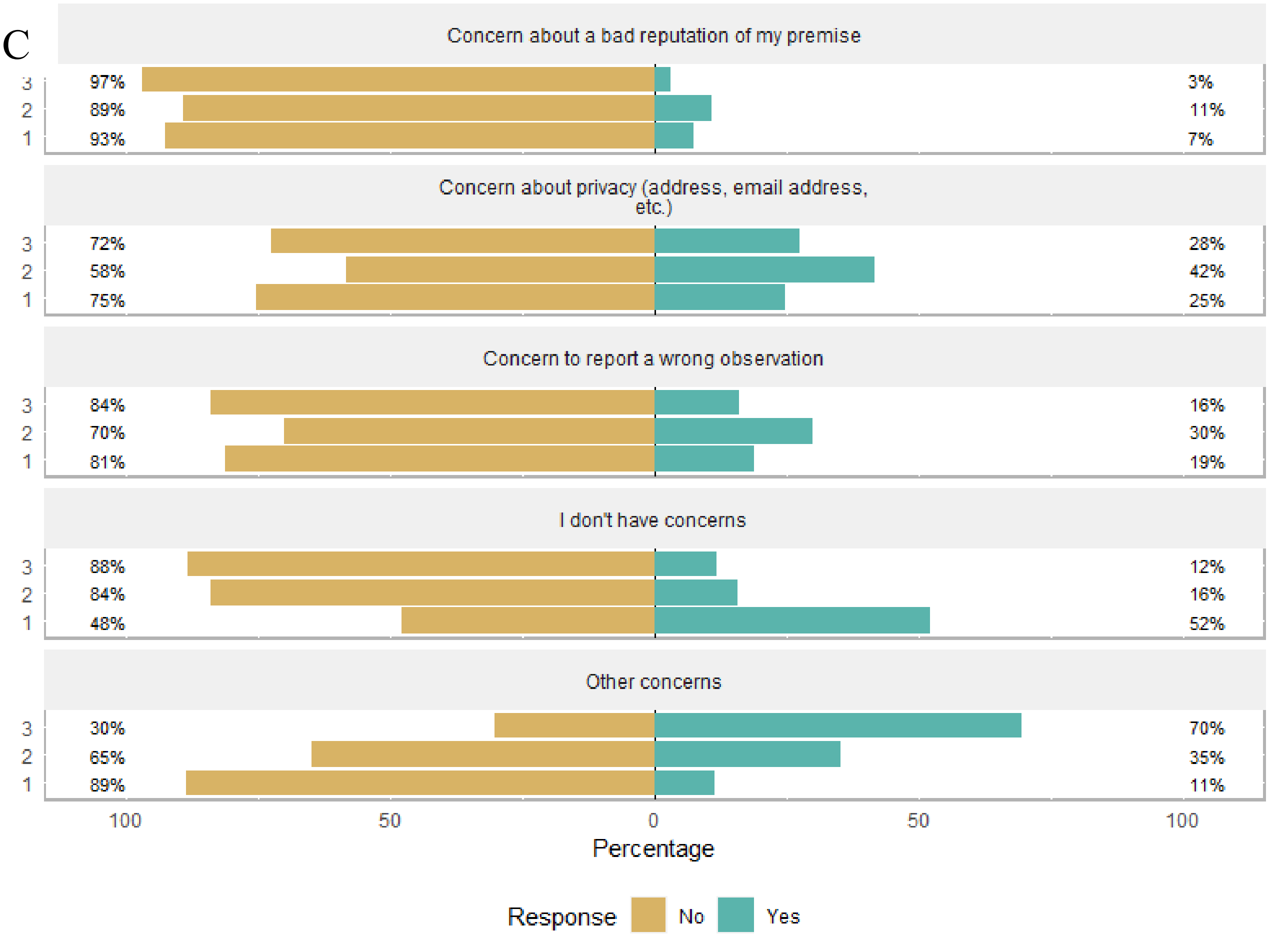

Supplementary figure 4 (A - C). Likert-scale answers given to the questions 21 – 23 from the online questionnaire, separated by attitude cluster 1 (highly positive attitude cluster), 2 (moderately positive attitude cluster) and 3 (negative attitude cluster).

3 Supplementary tables

Supplementary table 1. Questions included in the Multiple Component Analyses on the motivation and attitude of respondents towards community-based surveillance. Each sub question was considered as one variable with categorical answers.

Main question	Sub question	Answer scale
(21) Which of the following statements motivate you to report clinical signs of your equine on an online platform such as Equi-Commun?	I care about early detection of infectious diseases in my business because of financial reasons.<br>My reports are listed continuously on “Equi-Commun”. Therefore I get an overview about the reported health issues of the equine.<br>Receiving regularly newsletter with current information about “Equi-Commun” and about the current situation of equine diseases in Switzerland and foreign countries.<br>The insight to a map of Switzerland on which all registered reports are (anonymously) illustrated.<br>Receiving an e-mail in case of unusual increase in clinical signs in my region.<br>Receiving a text message in case of unusual increase in clinical signs in my region.	5 point Likert-scale (doesn’t motivate me at all – motivates me a lot) and the option doesn't apply to me
(22) How do you agree with the following statements?<br>“By observing and reporting clinical signs I support the early detection of infectious diseases and therefore …	… protect the health of my equine.”<br>… protect the health of equine on my premises.”<br>… protect the health of equine in my region.”<br>… protect the health of equine in Switzerland.”	5 point Likert-scale (I disagree – I agree)
(23) Which of the following would be reasons for you to not participate in Equi-Commun?	Concern about data security (address, e-mail address etc.)<br>Concern to report a wrong observation<br>concern about a bad reputation for my premises<br>None<br>Other reason	Multiple choice select (yes – no)

Supplementary table 2: Equi-Commun communication and advertisement actions

Conferences	Annual Epidemiology Conference of the German-speaking countries	September 2018
	Annual Swiss Equine Research Conference	April 2019
E-mail to participants of the online survey	Information on Equi-Commun launch	November 2018
	Christmas newsletter and reminder on Equi-Commun's launch	January 2018
Print media article	Report in the monthly Swiss equine magazine "Kavallo"	November 2018
	Report in the monthly Bulletin of the Swiss Equestrian Sports Association	November 2018
	Report in the weekly Swiss equine newspaper "Pferde Woche"	February 2019
Flyer distribution	As print outs - in equine clinics, veterinary practices, veterinary faculty cafeteria and FSVO<br>As online content - in newsletters and emails	Since November 2018
Facebook page Equi-Commun	Irregular social media posts	November 2018 to May 2019
Banner advertisement in online equestrian portal	Link to information webpage	Since July 2017
VPHI internal newsletter	Information distribution among the institute members	November 2018
Equinella newsletter	Information on Equi-Commun's launch to Equinella veterinarians	November 2018
Vetsuisse Bern veterinary faculty e-mail distributor	Information leaflet to Vetsuisse employees and students	January 2019
E-mail signature RÖ and SD	Equi-Commun Logo	Since December 2018
VPHI homepage subpage	Equi-Commun project description	Since January 2019
Communication with the head of the two University equine clinics	Personal meetings and hand-over of flyers	January 2019

Supplementary table 3. Four reports submitted to Equi-Commun

	Owner 1		Owner 2	Owner 3
	Report 1	Report 2	Report 3	Report 4
Date of submission	15.12.2018	15.12.2018	08.01.2019	09.02.2019
Date of clinical sign onset	03.09.2018	30.09.2018	03.01.2019	15.12.2018
Duration of the clinical sign (category)	≤ 3 days	> 2 weeks	unknown	> 2 weeks
Observed clinical sign category (category)	Colic	Lameness	Other (pastern dermatitis)	Lameness
Postal code and location	provided	provided	provided	provided
Equine name (free text)	provided	provided	provided	provided
Age (category)	> 4 years	> 4 years	> 4 years	> 4 years
Number of equine on premise	11 - 20	11 - 20	5 - 10	> 50
Veterinarian contacted?	yes	yes	no	yes
Veterinarian's diagnosis	Obstructive colic	Laminitis	-	Etiology of lameness unknown, possibly a dislocation or arthritis
Additional remark	Colic surgery at the equine hospital University of Zurich, 01.10.2018	Equine Metabolic Syndrome	-	
